# Lysophosphatidylcholine binds α-synuclein and prevents its pathological aggregation

**DOI:** 10.1093/nsr/nwae182

**Published:** 2024-05-25

**Authors:** Chunyu Zhao, Jia Tu, Chuchu Wang, Wenbin Liu, Jinge Gu, Yandong Yin, Shengnan Zhang, Dan Li, Jiajie Diao, Zheng-Jiang Zhu, Cong Liu

**Affiliations:** Interdisciplinary Research Center on Biology and Chemistry, Shanghai Institute of Organic Chemistry, Chinese Academy of Sciences, Shanghai 201210, China; University of Chinese Academy of Sciences, Beijing 100049, China; Interdisciplinary Research Center on Biology and Chemistry, Shanghai Institute of Organic Chemistry, Chinese Academy of Sciences, Shanghai 201210, China; University of Chinese Academy of Sciences, Beijing 100049, China; Interdisciplinary Research Center on Biology and Chemistry, Shanghai Institute of Organic Chemistry, Chinese Academy of Sciences, Shanghai 201210, China; University of Chinese Academy of Sciences, Beijing 100049, China; Interdisciplinary Research Center on Biology and Chemistry, Shanghai Institute of Organic Chemistry, Chinese Academy of Sciences, Shanghai 201210, China; Interdisciplinary Research Center on Biology and Chemistry, Shanghai Institute of Organic Chemistry, Chinese Academy of Sciences, Shanghai 201210, China; University of Chinese Academy of Sciences, Beijing 100049, China; Interdisciplinary Research Center on Biology and Chemistry, Shanghai Institute of Organic Chemistry, Chinese Academy of Sciences, Shanghai 201210, China; Interdisciplinary Research Center on Biology and Chemistry, Shanghai Institute of Organic Chemistry, Chinese Academy of Sciences, Shanghai 201210, China; Bio-X Institutes, Key Laboratory for the Genetics of Developmental and Neuropsychiatric Disorders, Ministry of Education, Shanghai Jiao Tong University, Shanghai 200030, China; Zhangjiang Institute for Advanced Study, Shanghai Jiao Tong University, Shanghai 200040, China; Department of Cancer Biology, University of Cincinnati College of Medicine, Cincinnati, OH 45267, USA; Interdisciplinary Research Center on Biology and Chemistry, Shanghai Institute of Organic Chemistry, Chinese Academy of Sciences, Shanghai 201210, China; Interdisciplinary Research Center on Biology and Chemistry, Shanghai Institute of Organic Chemistry, Chinese Academy of Sciences, Shanghai 201210, China

**Keywords:** α-synuclein, Parkinson's disease, lysophosphatidylcholine, aggregation

## Abstract

Accumulation of aggregated α-synuclein (α-syn) in Lewy bodies is the pathological hallmark of Parkinson's disease (PD). Genetic mutations in lipid metabolism are causative for a subset of patients with Parkinsonism. The role of α-syn's lipid interactions in its function and aggregation is recognized, yet the specific lipids involved and how lipid metabolism issues trigger α-syn aggregation and neurodegeneration remain unclear. Here, we found that α-syn shows a preference for binding to lysophospholipids (LPLs), particularly targeting lysophosphatidylcholine (LPC) without relying on electrostatic interactions. LPC is capable of maintaining α-syn in a compact conformation, significantly reducing its propensity to aggregate both *in vitro* and within cellular environments. Conversely, a reduction in the production of cellular LPLs is associated with an increase in α-syn accumulation. Our work underscores the critical role of LPLs in preserving the natural conformation of α-syn to inhibit improper aggregation, and establishes a potential connection between lipid metabolic dysfunction and α-syn aggregation in PD.

## Teaser

α-Synuclein (α-syn) was found to preferentially bind lysophospholipids (LPLs), particularly targeting lysophosphatidylcholine (LPC). LPC plays a critical role in preserving the natural conformation of α-syn and inhibiting pathological aggregation of α-syn.

## INTRODUCTION

Abnormally aggregated α-synuclein (α-syn) is a major component of Lewy bodies, the pathological hallmark of familial and sporadic Parkinson's disease (PD) and other synucleinopathies [1–3]. α-Syn is intrinsically disordered and can self-assemble into amyloid aggregates under pathological and *in vitro* experimental conditions [4]. Despite its high propensity to aggregate, native α-syn is highly abundant in neurons (∼1% of all proteins) and resists aggregation in normal intracellular environments [5–8]. How native α-syn maintains its conformation is a long-standing puzzle and fundamental to our understanding of the physiological function of α-syn and the molecular mechanisms underlying α-syn aggregation.

Genetic defects in lipid metabolism have been identified in association with a subset of Parkinsonism characterized by α-syn aggregation and Lewy-body formation [3,9–11]. α-Syn contains a putative lipid-binding domain. Extensive studies have been applied to investigate the interactions between α-syn and membranes. α-Syn can bind to a variety of artificial lipid and detergent assemblies *in vitro* (e.g. phospholipid (PL), lysophosphatidylglycerol (LPG), sodium dodecyl sulfate (SDS) and fatty acid (FA)) [12–15]. However, the influence of lipid binding to α-syn is complex as some lipids induce α-syn fibril self-assembly, while others inhibit it [16–18]. Systematically characterizing the lipid binding profile of native α-syn is important not only to understand its physiological function, but also its pathological activity in PD.

Here, we describe a mass-spectrometry-based untargeted profiling study to identify the lipid binder(s) of α-syn. We found that both endogenous α-syn and recombinant α-syn monomers preferentially bind lysophospholipids (LPLs), especially lysophosphatidylcholine (LPC). We further demonstrated that LPC can induce the conformational transition of α-syn independent of electrostatic interaction, and significantly inhibits α-syn pathological aggregation, while reduced production of LPC promotes α-syn aggregation. This work provides a comprehensive analysis of interactions between α-syn and LPC and highlights the involvement of lipid metabolism and lipid–protein interaction in neurodegenerative diseases.

## RESULTS

### The α-syn monomer preferentially binds to LPLs

**Figure 1.**
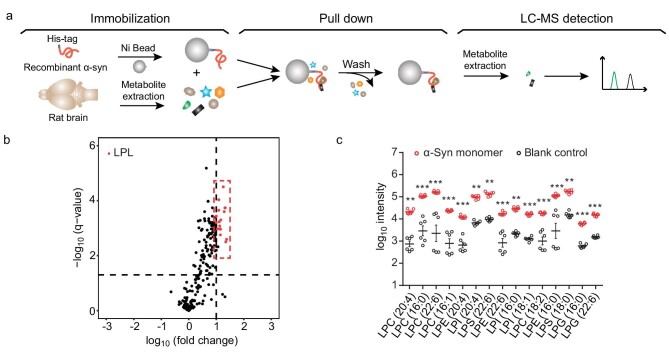
The α-syn monomer shows the preference for binding to LPLs *in vitro*. (a) Schematic illustration of the *in vitro* metabolite profiling assay developed in this study. His-tagged α-syn monomers were immobilized on Ni beads and incubated with metabolite extracts from rat brains. Bound metabolites were detected and identified by LC-MS-based metabolite profiling. (b) Volcano plots of the identified metabolites. LPLs that bind with the α-syn monomer with high significance and fold change are highlighted in red. The horizontal dashed line indicates a q-value of 0.05. The vertical dashed line indicates a fold change of 10. q-Values were calculated by Student's t-test followed by false discovery rates (FDR) correction. Fold change represents the metabolite intensity of the α-syn sample over that of the blank sample. (c) Intensities of the top-ranking LPLs that bind with the α-syn monomer in comparison with those of the blank control. Data represent the mean ± SEM (standard error of mean) (*n* = 6). *, q-value < 0.05; **, q-value < 0.01; ***, q-value < 0.001; Student's t-test followed by FDR correction.

To profile the metabolite binding partners for the α-syn monomer, we developed an *in vitro* assay using liquid chromatography-mass spectrometry (LC-MS)-based untargeted profiling. The α-syn monomers were immobilized on Ni-sepharose beads and incubated with metabolite mixtures extracted from rat brains and SH-SY5Y cells, respectively. The bound metabolites were eluted and analyzed by LC-MS/MS (Fig. 1a). The lipids were further identified by searching against a comprehensive lipid MS/MS spectral library that we established previously, including 76 361 lipid species and 181 300 predicted MS/MS spectra, representing 34 lipid classes [19]. In addition, an in-house metabolite MS/MS spectral library derived from 843 standards and a commercially available database NIST17 were also involved for metabolite identification. nsLTP, a plant non-specific lipid transfer protein, is used as a positive control, showed a preference for lipids, including PLs, LPLs and polyunsaturated fatty acid (PUFA) (Fig. S1a and b). Remarkably, we found that α-syn monomers, but not the negative control protein (TrpEG), pulled down a variety of lipids with a strong binding preference to the lyso form of glycerophospholipids (GPs)—LPLs (Figs 1b and c, S1c and S2 and Table S1). In addition, a similar metabolite-binding profile of the α-syn monomer was obtained by using metabolite mixtures prepared from both rat brains and human SH-SY5Y cells (Tables S1 and S2), implying the robust lipid binding profile of the α-syn monomer to LPLs across different metabolite resources. Of note, we extracted lipid/metabolite with MeOH/ACN/H_2_O solvents which prefer extraction of polar and medium hydrophobic lipids [20–22]. Thus, highly hydrophobic lipids (e.g. triacylglycerols) may be overlooked in our *in vitro* pull-down assays.

### LPLs are associated with α-syn in cells and in rat brains

**Figure 2.**
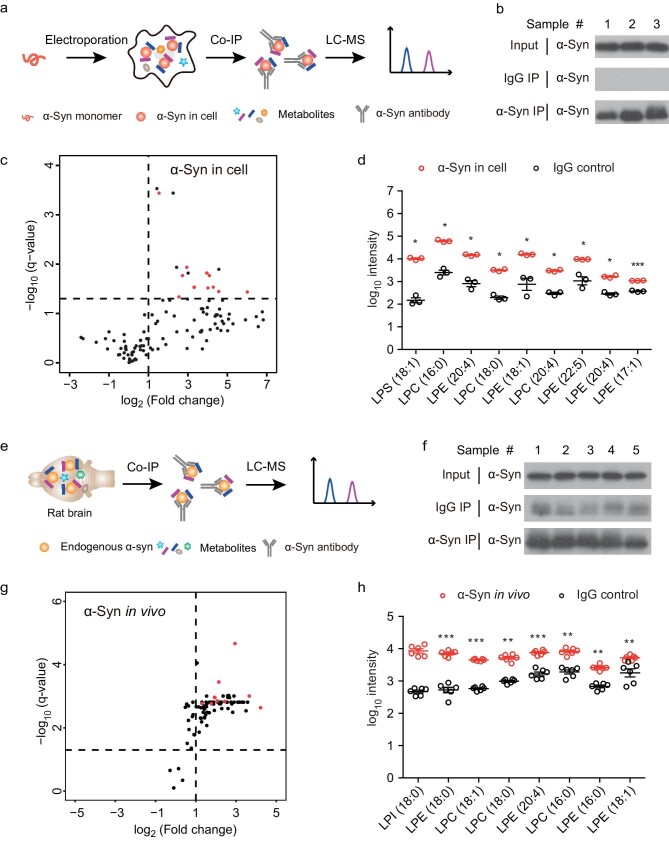
LPL interacts with α-Syn in cells and *in vivo*. (a, e) Schematic illustration of the metabolite profiling for in-cell α-syn (a) and *in vivo* α-syn (e). Recombinant α-syn monomers were electroporated into HEK 293T cells and then immunoprecipitated by α-syn antibody (a). Endogenous α-syn in rat brains was extracted by immunoprecipitation (e). Co-precipitated metabolites were identified by LC-MS. (b, f) Western blot confirmed that α-syn was extracted from cells (b) and endogenous α-syn was extracted from the rat brain (f) by IP. (c, g) Volcano plots show the metabolite-binding profile of in-cell α-syn (c) and endogenous α-syn (g). LPLs with significant changes (fold change > 2, q-value < 0.05) are highlighted in red. Fold change represents the metabolite intensity of the α-syn sample over that of the IgG control sample. (d, h) Intensity of the top-ranking LPLs that co-precipitated with in-cell α-syn (d) and endogenous α-syn (h). Data represent the mean ± SEM (*n* = 3 in d, *n* = 6 in h), *, q-value < 0.05; **, q-value < 0.01; ***, q-value < 0.001; Student's t-test followed by FDR correction.

The experiments described above profiled the metabolite ligands that bind to α-syn in a simplified *in vitro* environment where only α-syn and metabolites presented. Next, we sought to identify the metabolites that bind to α-syn in cells. We transfected HEK 293T cells with monomeric α-syn by electroporation, followed by extracting the transfected α-syn with α-syn antibody by immunoprecipitation (IP) (Fig. 2a and b). Metabolites that co-immunoprecipitated with α-syn were identified by LC-MS-based profiling as described in the *in vitro* assay. Consistently, in the complex intracellular environment, α-syn also showed a preference for a wide spectrum of LPLs over other lipids (Fig. 2c and d and Table S3), similar to that observed in the *in vitro* pull down with α-syn monomers (Fig. 1b and c and Table S1).

To further explore the binding preference of α-syn in its native conformation and environment, we profiled the metabolite binders of endogenous α-syn in the rat brain. Endogenous α-syn was isolated by IP from rat brains (Fig. 2e and f). By using LC-MS-based profiling, again, we found many subclasses of LPLs that are in association with endogenous α-syn, similar to what was observed with recombinant α-syn in the *in vitro* and in-cell pull-down assays (Fig. 2g and h and Table S4). In addition, endogenous α-syn was also identified as binding with PLs (Table S4), especially phosphatidylserine (PS), which has been widely used to study the α-syn–lipid interaction *in vitro* [16]. The metabolite profiling of α-syn in the rat brain demonstrates that endogenous α-syn in the central nervous system (CNS) also prominently forms complexes with LPLs, indicating the biological relevance of LPLs’ association with α-syn. Notably, from both *in vitro* and *in vivo* pull-down assays, LPCs represent the largest subfamily of LPLs preferentially binding to α-syn monomers.

Since our *in vivo* profiling data described above suggest that native α-syn has a strong preference for LPLs, we further conducted quantitative lipidomic profiling of mouse brains and synaptic vesicles (SVs) to explore the intracellular distribution of LPLs in the brains, which will help to understand the biological significance of the interaction between LPLs and α-syn. We extracted the lipids from mouse brains and performed absolute quantitative lipidomic profiling. We found that LPLs represent a minor class of lipids with an occupancy of ∼0.73% in the total GPs of mouse brains (Fig. S3a, Table S5). Among LPLs, five major species were identified including lysophosphatidylethanolamine (LPE) (95.0% of all LPLs), LPC (4.2%), lysophosphatidylserine (LPS) (0.6%), lysophosphatidic acid (LPA) (0.1%) and lysophosphatidylglycerol (LPG) (0.1%) (Fig. S3a). Note that LPE and LPC are the most abundant species of LPLs and both are zwitterionic. We next purified SVs from mouse brains (Fig. S4). Lipids were extracted from purified SVs, followed by absolute lipid quantification. The result showed that the population of the majority of GPs, including PLs and LPLs, in the SV membrane showed no significant difference from that of the total brain membranes. One remarkable exception was LPC, the second most abundant species of LPL, which was enriched in SVs: the percentages of LPC in all LPLs of brains and SVs were 4.2% and 21.8%, respectively (Fig. S3b). To further profile the distribution of LPLs in the SV membrane, we partitioned the SV membrane into 13 fractions by sucrose gradient sedimentation (Fig. S3c), and analyzed their lipid compositions by LC-MS. It has been reported that lipid rafts are rich in cholesterol [23]. Our result showed that cholesterol is populated in fraction 3, which, together with the western blot analysis, indicates that lipid rafts are dominantly presented in fraction 3 (Fig. S3c and d). Notably, LPCs, but not PLs, are also enriched in fraction 3 (Fig. S3d). In particular, LPCs that contain saturated fatty acyl chains, such as LPC (16 : 0) and LPC (18 : 0), are significantly enriched in this fraction (Fig. S3d). Lipid rafts are micro-domains of functional lipids and proteins coalescing in membranes [24,25]. Thus, despite the low abundance of LPC (0.16%) in the GPs of the SV membrane, LPC in lipid rafts may present with a relatively high local concentration. As we measured, the amount of LPC in the lipid raft fraction is 1.35 ± 0.08 pmol/µg, while in non-raft fractions it is 0.82 ± 0.04 pmol/µg protein. Since α-syn also presents in the lipid rafts of SVs as we identified (Fig. S3c) and previously reported [26], LPC may cooperate with α-syn in the lipid rafts of the SV membrane and play an important role in α-syn function. Indeed, we recently demonstrated that LPC exhibits high activity in promoting α-syn-induced vesicle clustering, a key step in neuronal SV trafficking [27]. Thus, we next focus on the interaction between α-syn and LPC.

### Characterization of LPC–α-syn interaction

To investigate the mechanistic basis of α-syn binding to LPC, we used multiple biophysical techniques. First, to distinguish the form of LPC that binds with α-syn, we performed native MS, which is a robust tool for the analysis of non-covalent complexes whereby the biological samples are analyzed in a non-denaturing solvent. The result showed that LPC in the form of a micelle (a spherical self-assembled surface of lipids), but not monomer, binds with α-syn (Fig. S5). This result suggests that α-syn binds to the membrane surface arranged by LPC. We thus used LPC micelles in subsequent studies of LPC–α-syn interaction.

**Figure 3.**
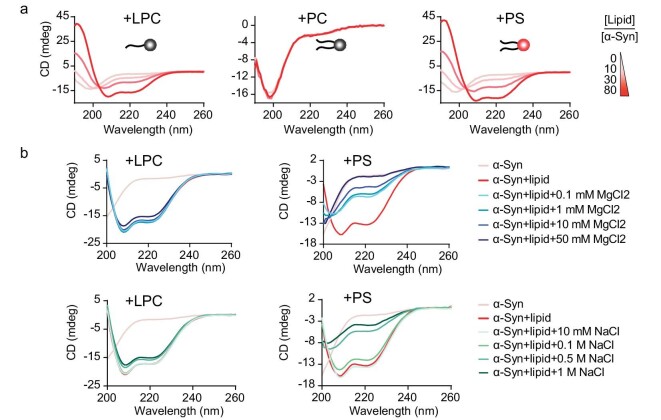
LPC induces the structural transition of α-syn independent of electrostatic interaction. (a) The secondary structure transition of α-syn in the presence of LPC micelles, PC liposomes and PS liposomes monitored by circular dichroism (CD) spectroscopy. Lipid molecules are shown as cartoons with neutral head groups (in the left and middle panels) and negatively charged head groups (in the right panel). The color bar indicates the molar ratios of lipid/α-syn. (b) CD spectroscopy shows that as concentrations of salts (MgCl_2_ or NaCl) increase, the DOPS–α-syn interaction was disrupted accordingly, whereas the LPC–α-syn interaction was not apparently affected.

Previous reports have shown that in the presence of negatively charged lipids such as dioleoylphosphatidylserine (DOPS), but not dioleoylphosphatidylcholine (DOPC) that is zwitterionic, α-syn undergoes the conformational transition from random coil to α-helix [28,29]. Interestingly, we found that LPC, which carries a choline group, a phosphate unit and a single fatty acyl chain, could bind to α-syn with an affinity comparable to that of electronegative DOPS, and induced the α-helix-rich conformation of α-syn (Fig. 3a). More importantly, unlike the DOPS-α-syn interaction, which could be disrupted by the addition of salts such as MgCl_2_, the interaction of LPC–α-syn was resistant to increasing concentrations of salts (Fig. 3b).

### LPC induces and stabilizes the compact structure of α-syn

**Figure 4.**
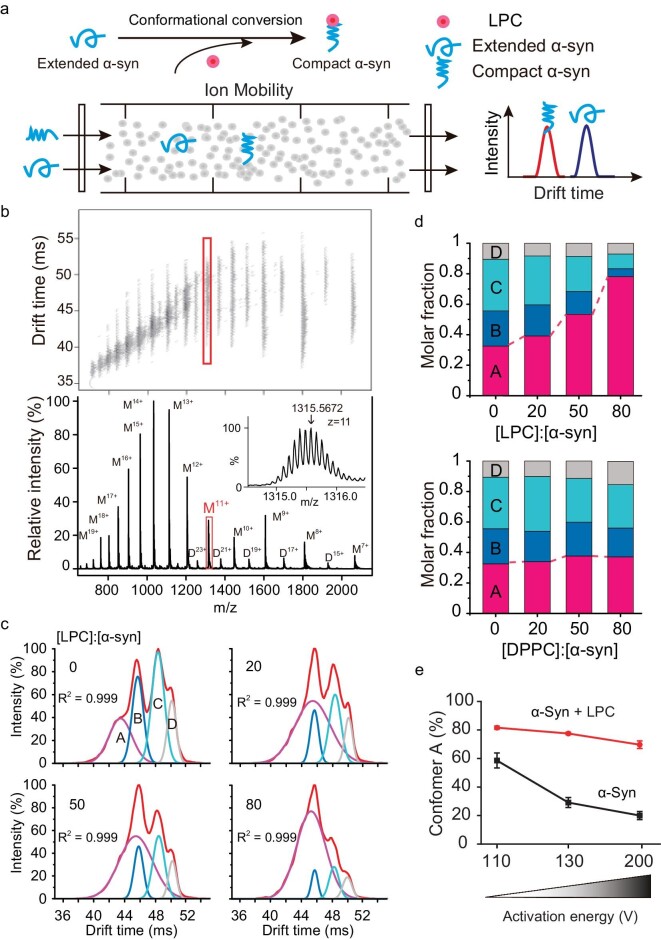
LPC compacts α-syn and increases its stability. (a) The schematics of IM-MS technology. (b) IM-MS spectrum of α-syn with charge-state distributions. Monomeric charged state (11^+^) is highlighted with a frame, and is zoomed in with labels in the inset. (c) IM-MS spectrum isolated from 11^+^ charged α-syn was deconvolved into four conformers (A–D) on the basis of Ω acquired from α-syn in the presence of LPC. (d) Population distribution change of the four conformers (A–D) upon the addition of LPC (upper) and DPPC (lower). (e) The compact conformer A of α-syn is stabilized by LPC and resists activation-induced unfolding.

To understand the mechanism of the interaction between α-syn and LPC, we applied ion mobility-mass spectrometry (IM-MS) to characterize the structural conversion and stability of α-syn in the presence of LPC. IM-MS simultaneously measures the rotationally averaged collision cross-sections (CCS) of multiple ion populations of a protein in the gas phase (Fig. 4a), and provides unique advantages in probing protein and lipid interactions [30], especially for amyloid proteins which feature highly structural heterogeneity [31,32]. In our work, predominantly monomer-related ions (from 7^+^ to 20^+^) together with traces of dimer ions were detected for α-syn (Fig. 4b). As an example, monomeric α-syn charge state (11^+^) was mainly populated with four conformers and distinct CCS values represented by the most compact (Ω ∼2775.0 Å^2^, conformer A) to extended (Ω ∼3298.6 Å^2^, conformer D) structures (Fig. 4b and c). Notably, the addition of LPC rather than dipalmitoylphosphatidylcholine (DPPC) resulted in the population redistribution of α-syn towards the most compact conformer A, the proportion of which significantly increased from 30% to 80% (Figs 4c and d and S6a). This result suggests that the increased levels of LPC are able to drive the conformational conversion of α-syn from relatively unstructured conformers to compact conformer A.

To investigate the impact of LPC on the stability of α-syn, we next applied an activation-induced unfolding strategy in IM-MS to monitor the conformational change of conformer A upon LPC binding. The result showed that conformer A gradually unfolded into extended conformers as the activation energy increased in the absence of LPC. In contrast, in the presence of LPC, conformer A showed a significantly enhanced resistance to unfolding with elevated activation voltage (Figs 4e and S6b). Taken together, our results indicate that LPC can promote and stabilize α-syn in a compact, stable structure, which certifies the vital role of LPC in preserving the soluble form of α-syn and protecting α-syn from amyloid aggregation.

### LPC protects α-syn from aggregation

**Figure 5.**
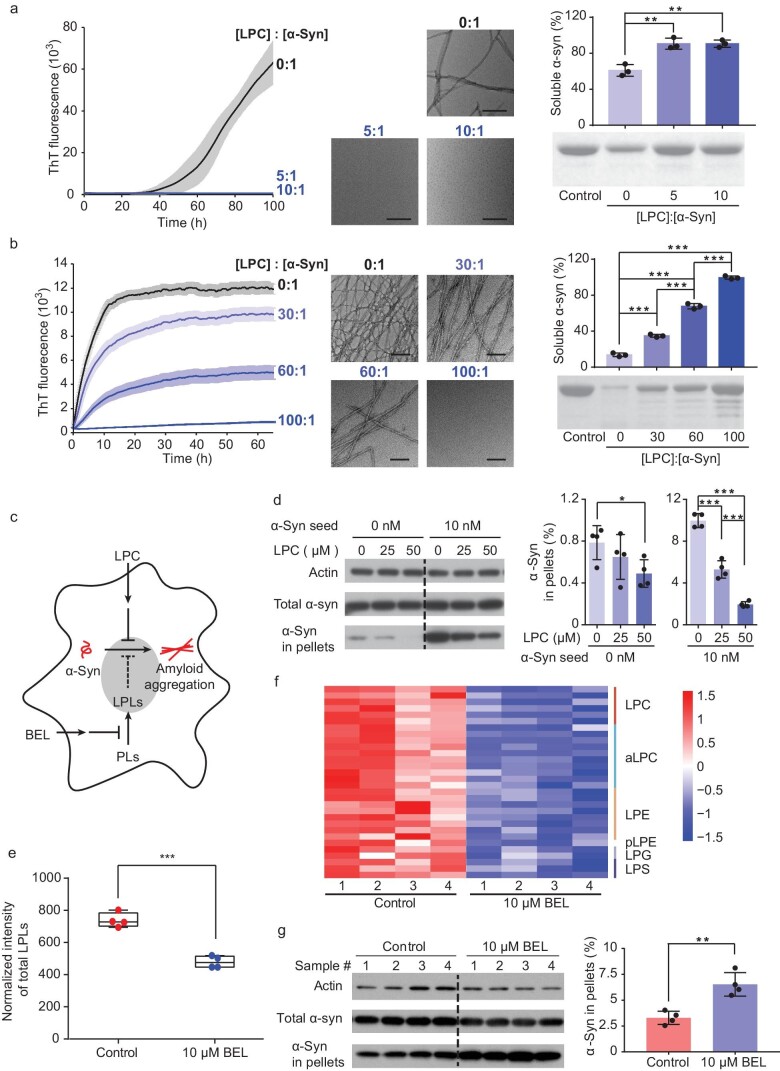
LPC inhibits α-syn aggregation *in vitro* and in cells. (a, b) The ThT kinetic assay (graph on left) and the analysis of soluble α-syn (graph on right) shows the inhibition effect of LPC on α-syn aggregation at relatively low LPC/α-syn ratios (a) and high LPC/α-syn ratios (b). α-Syn aggregation was enhanced by the addition of 0.05% (v/v) preformed α-syn fibril seeds in (b). Transmission electron microscopy (TEM) images in the middle were taken at the end of the ThT assay for each sample. Scale bar = 200 nm. Data represent the mean ± SD (*n* = 3). **, *P*-value < 0.01; ***, *P*-value < 0.001; Student's t-test. (c) Schematic illustration of the cellular experiments. Chemical inhibitor BEL was added to inhibit the phospholipase that converts PLs to LPLs, to inhibit the production of LPLs. Reduced LPL production may impair the protection of LPLs against α-syn aggregation. (d) Western blot and the analysis of α-syn in-cell pellets show a dose-dependent inhibition of intracellular α-syn aggregation by treating cells with LPC. Preformed α-syn fibril seeds were used to promote α-syn aggregation. Data represent the mean ± SD (*n* = 4). *, *P*-value < 0.05; ***, *P*-value < 0.001; Student's t-test. (e) Normalized intensity of total LPL amounts in the control cells and BEL-treated cells. The total amounts of LPLs were calculated using the sum of Z-score-normalized (x/standard deviation) intensity for each LPL. ***, *P*-value < 0.001; Student's t-test. (f) Heat map of the lipidomic profiling of BEL-treated cells. LPLs with a significant change in amount (q-value < 0.05) are presented and colored by Z-scores. The lipid species within each cluster are shown in the right column. pLPE is a 1Z-alkenyl ether (plasmalogen) substituent of LPE. aLPC is an alkyl ether substituent of LPC. Four biological replicates for each sample were measured. Student's t-test followed by FDR correction. (g) Western blot and the analysis of α-syn in-cell pellets show that in the presence of BEL, insoluble α-syn aggregates dramatically increased compared to those in the control. Data are shown as means ± SD (*n* = 4). **, *P*-value < 0.01; Student's t-test.

To investigate the pathophysiological significance of LPC binding to α-syn, we conducted ThT kinetic assays to inspect the influence of LPC on α-syn amyloid aggregation at a series of lipid/protein ratios. The result showed that from low LPC/α-syn ratios (5 : 1 and 10 : 1) to high ratios (up to 100 : 1), with or without seeding the amyloid formation process, LPC persistently and effectively inhibited α-syn aggregation (Fig. 5a and b). We further tested the effect of LPC on α-syn aggregation in the intracellular environment (Fig. 5c). We first added an increasing amount of α-syn fibril seeds in the cell culture to enhance the aggregation of intracellular α-syn in the presence of different amounts of LPC. We found that the addition of LPC alleviated the aggregation of intracellular α-syn in a concentration-dependent manner without affecting the uptake of fibril seeds (Figs 5d and S7). Conversely, we applied BEL, a chemical inhibitor of calcium-independent phospholipase A2 (iPLA2), to inhibit the production of LPLs [33], which led to a reduction in the levels of LPLs, including nearly 40 species of LPLs (Fig. 5e and f and Table S6). We found that the presence of BEL led to a significant enhancement of α-syn aggregation (Fig. 5g). Together, these results indicate the role of LPC in protecting α-syn from amyloid aggregation.

Furthermore, we observed that the familial PD mutation of A30P in the lipid binding domain resulted in a 10-fold decrease in the binding affinity of α-syn with LPC, while the other known familial mutations, i.e. E46K, H50Q, G51D and A53T/E, which directly affect the amyloid fibril structure of α-syn [34], showed minimal influence on the LPC-binding affinity of α-syn (Fig. S8a). Accordingly, the inhibitory effect of LPC on the aggregation of the A30P mutant is significantly weaker than that of the wild-type and other mutants, e.g. G51D (Fig. S8b). The impact of A30P mutation in weakening the binding of α-syn with LPC supports the pathophysiological significance of α-syn–LPC interaction in mediating the native function of α-syn and the pathogenesis of PD.

## DISCUSSION

In this study, we discovered that the α-syn monomer has a preference for binding to LPLs and provided insights into how the interaction with LPC might prevent α-syn aggregation. A critical balance between LPC levels and α-syn may be essential for α-syn's physiological functions, with disturbances in this equilibrium, such as α-syn overexpression, mutations or lipid homeostasis disruption, potentially leading to Parkinsonism-related pathology. Notably, a previous study revealed that LPC levels were reduced in the primary visual cortex region of human PD tissues [35], supporting this notion. Moreover, missense mutations in the Phospholipase A2 Group VI (*PLA2G6*) gene are causative to multiple neurodegenerative diseases, including INAD (infantile neuroaxonal dystrophy), NBIA (idiopathic neurodegeneration associated with brain iron accumulation) and PD14 [9–11,36]. Intriguingly, gene *PLA2G6* encodes calcium-independent phospholipase A2 (iPLA2), a phospholipase that catalyzes the conversion of PLs to LPLs and PUFAs [33]. Thus, the *PLA2G6* mutation may interfere with LPL production and may impair the interaction of LPLs with α-syn in regulating α-syn normal function and pathological aggregation. Indeed, as we inhibited iPLA2 in cells, we observed a significant decrease in LPLs and enhanced α-syn aggregation in cell models. The accumulation of α-syn aggregation has also been observed in the brains of patients with *PLA2G6* mutations and *PLA2G6*^−/−^ mice [3,37]. This suggests that *PLA2G6* mutations directly contribute to α-syn aggregation and neurodegeneration, providing significant insights into the interaction of endogenous LPC with α-syn in the development of PLA2G6-associated neurodegeneration (PLAN) and general neurodegeneration.

In contrast to earlier research emphasizing the significance of negative surface charge in the interaction between α-syn and lipids [28,29,38,39], our work shows that LPC, a key endogenous lipid binder for α-syn, is zwitterionic. The binding between α-syn and LPC is not influenced by electrostatic interactions and is unaffected by changes in salt concentrations. This finding differs from the behavior observed with negatively charged lipids, such as PS, whose α-syn binding is sensitive to salt levels and may not be compatible with the natural salt concentrations found in neurons. Furthermore, LPC is characterized by its unique inverted-cone shape and a single fatty acyl chain, leading to looser lipid packing and increased curvature [40–42]. These properties are likely to enhance α-syn's hydrophobic interaction with synaptic vesicle membranes, aiding in α-syn's role in synaptic vesicle trafficking within neurons. Moreover, α-syn was recently found to undergo liquid-liquid phase separation which was believed to recruit SVs in the condensates for dynamic regulation of SV trafficking [43,44]. The interplay between α-syn and LPC may also be involved in modulating this process, something which needs further investigation.

## MATERIALS AND METHODS

For detailed materials and methods, please see the Supplementary Data. The animal experiments followed the protocols approved by the Animal Care Committee of the Interdisciplinary Research Center on Biology and Chemistry, Chinese Academy of Sciences. Detailed materials and methods are available in the supplementary data.

## Supplementary Material

nwae182_Supplemental_Files
