# Supplementary material for: Lysophosphatidylcholine binds α-synuclein and prevents its pathological aggregation
Source: Natl Sci Rev. 2024 May 25;11(6):nwae182. doi: 10.1093/nsr/nwae182 (PMC11221426; doi:10.1093/nsr/nwae182)
Supplement: nwae182_Supplemental_Files [file nwae182_supplemental_files.zip › nwae182-Supplementary Materials_revision-0620-no highlight.pdf]

Supplementary Materials for

Lysophosphatidylcholine binds α-synuclein and prevents its pathological aggregation

Chunyu Zhao[1,2#], Jia Tu[1,2#], Chuchu Wang[1,2#], Wenbin Liu[1], Jinge Gu[1,2], Yandong Yin[1], Shengnan Zhang[1], Dan Li[3], Jiajie Diao[4*], Zheng-Jiang Zhu[1*], Cong Liu[1*]

[1]Interdisciplinary Research Center on Biology and Chemistry, Shanghai Institute of Organic Chemistry, Chinese Academy of Sciences, Shanghai 201210, China;

[2]University of Chinese Academy of Sciences, Beijing 100049, China;

[3]Bio-X Institutes, Key Laboratory for the Genetics of Developmental and Neuropsychiatric Disorders, Ministry of Education, Shanghai Jiao Tong University, Shanghai, 200030, China;

[4]Zhangjiang Institute for Advanced Study, Shanghai Jiao Tong University, Shanghai, 200040, China;

[5]Department of Cancer Biology, University of Cincinnati College of Medicine, Cincinnati, Ohio 45267, USA.

[#] These authors contributed equally to this work.

[*]To whom correspondence should be addressed. E-mails: liulab@sioc.ac.cn, jiangzhu@sioc.ac.cn, jiajie.diao@uc.edu

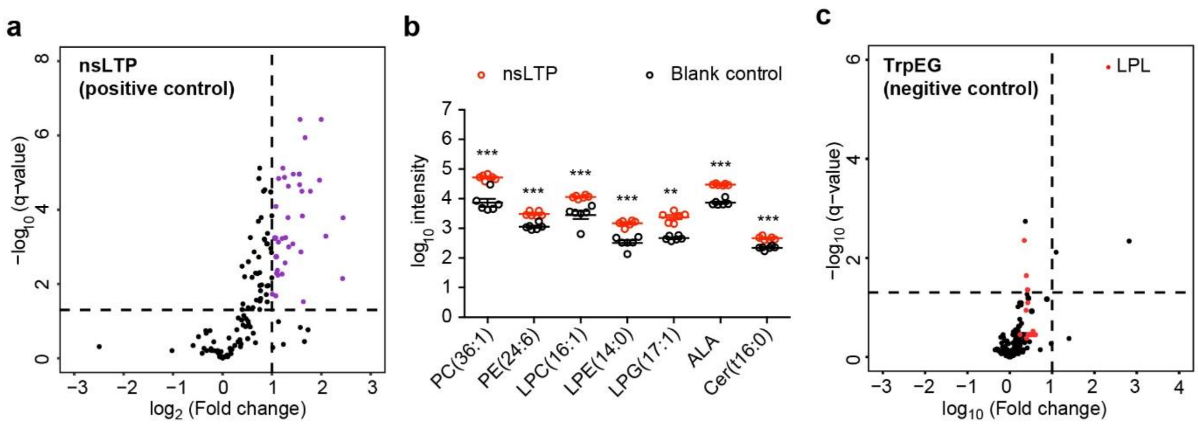

Figure S1. Metabolite-binding profiles of control proteins by the *in vitro* pull-down experiment.

a, Volcano plot of the identified metabolites that were pulled down by nsLTP. nsLTP is plant non-specific lipid transfer protein, which is known to bind a wide range of lipids and thus used as a positive control to validate the profiling assay. Purple dots highlight the identified metabolites with a fold change >2 and q-value < 0.05. The horizontal and vertical dash lines represent q-value of 0.05 and fold change of 2, respectively.

b, Intensity of the lipids bound to nsLTP in comparison with that of the blank control. Each lipid was one of the lipids from its subclass significantly bound to nsLTP. Data represents the mean ± SEM (n=6). **, q-value < 0.01; ***, q-value < 0.001; Student's t-test followed by FDR correction.

c, Volcano plot showed the metabolites profiles of TrpEG *in vitro*. TrpEG was used as a negative control, which is not a lipid-binding protein. Red dots highlight the LPLs that preferentially bind with α-syn monomer. The horizontal and vertical dash lines represent q-value of 0.05 and fold change of 10, respectively.

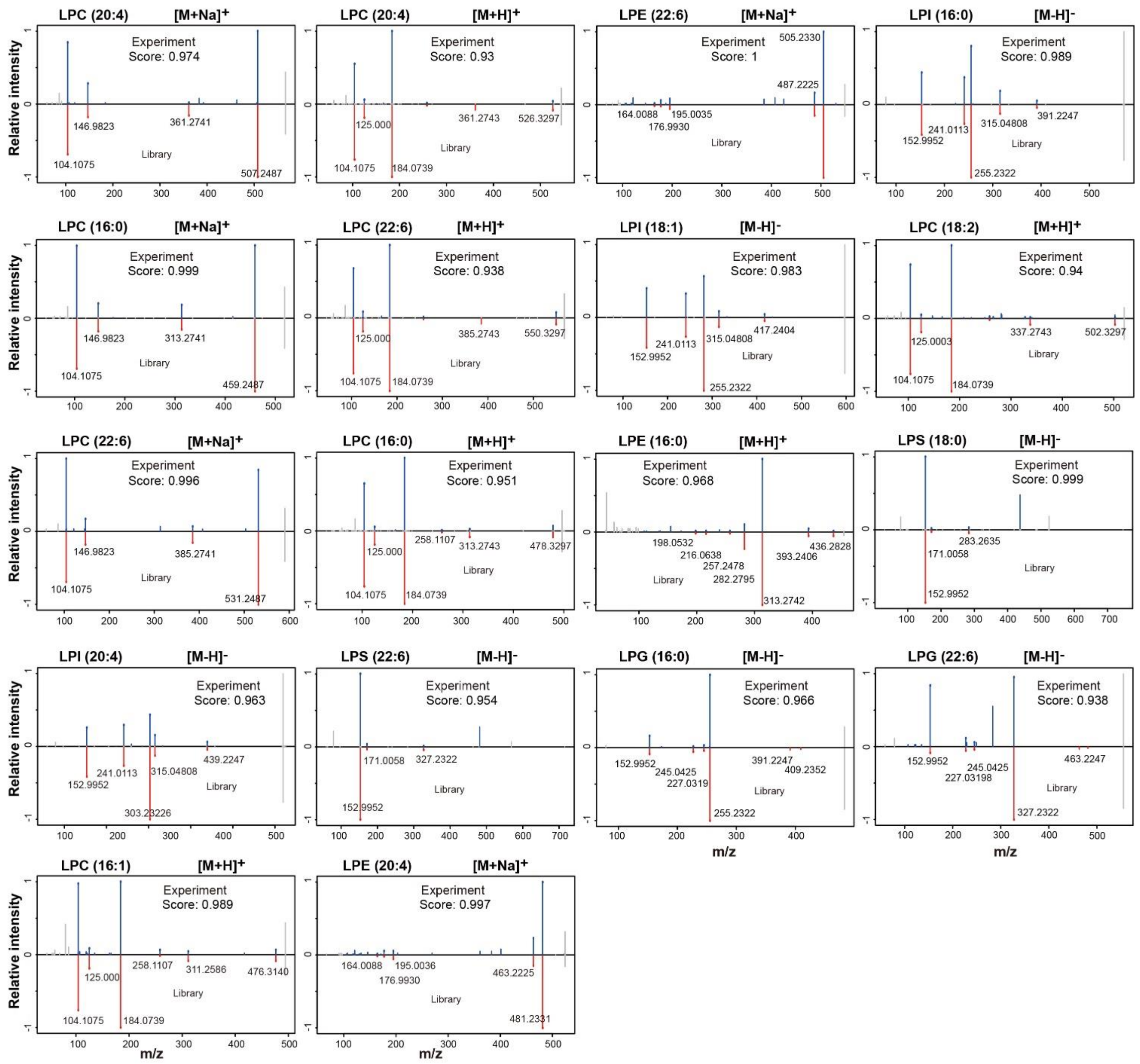

Figure S2. Identification of the top-ranking LPLs that bind with α-syn monomer by MS/MS spectral match.

Each box represents the MS/MS spectrum match of the experimental spectrum (blue) with reference library (red) of lipid ion. The MS/MS spectral similarity score are displayed in each box.

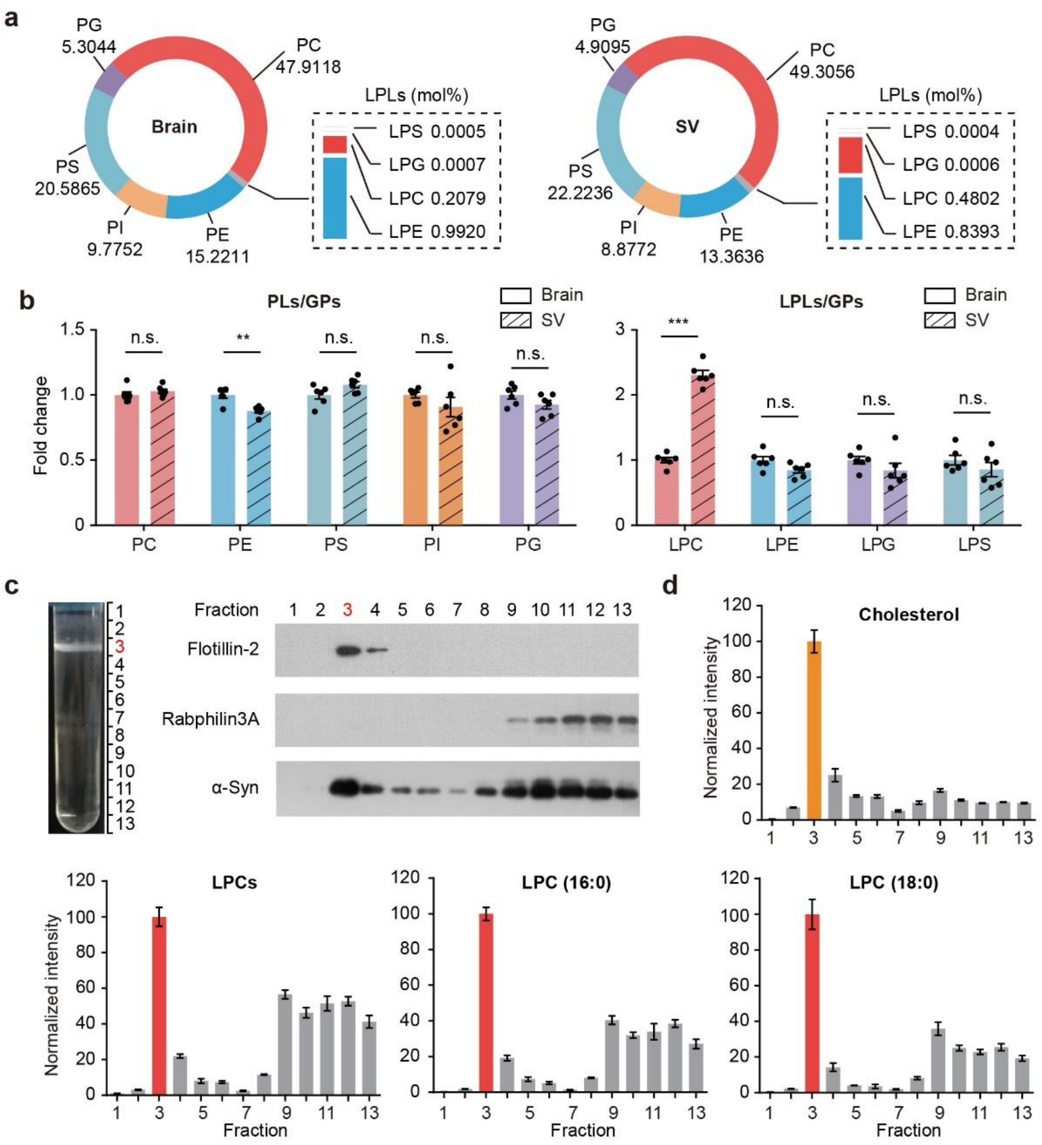

Figure S3. Quantitative lipidomic profilings for mouse brain and SV.

a, Quantitative profiling for GPs in the mouse brain and SVs.

b, Comparison of the percentage of different GPs including PLs and LPLs in the brain and SVs. Fold change was calculated according to the mole percentage of lipids in GPs with the brain lipids as 1.

c, Fractionation of SV membrane. The SV membrane was fractionated into 13 fractions by sucrose gradient sedimentation. The fractions were immunoblotted for Flotillin-2 (positive marker of lipid rafts), Rabphilin 3A (negative marker of lipid rafts) and α-syn. The enrichment of Flotillin-2, but not Rabphilin 3A in fraction 3 indicates that the lipid rafts dominantly exist in fraction 3.

d, LPC distribution in SV membrane. LPC, especially LPC (16:0) and LPC (18:0), are enriched in lipid rafts (fraction 3). Cholesterol was used as a positive control which is known to enrich in lipid rafts. Normalized intensities were calculated based on the peak area with the intensity of fraction 3 as

100. Data in (**b** and **d**) represent the mean ± SEM (n=6 in **b**; n=3 in **d**). *, *p*-value < 0.05; **, *p*-value < 0.01; ***, *p*-value < 0.001; n.s. represents "not significant"; Student's t-test.

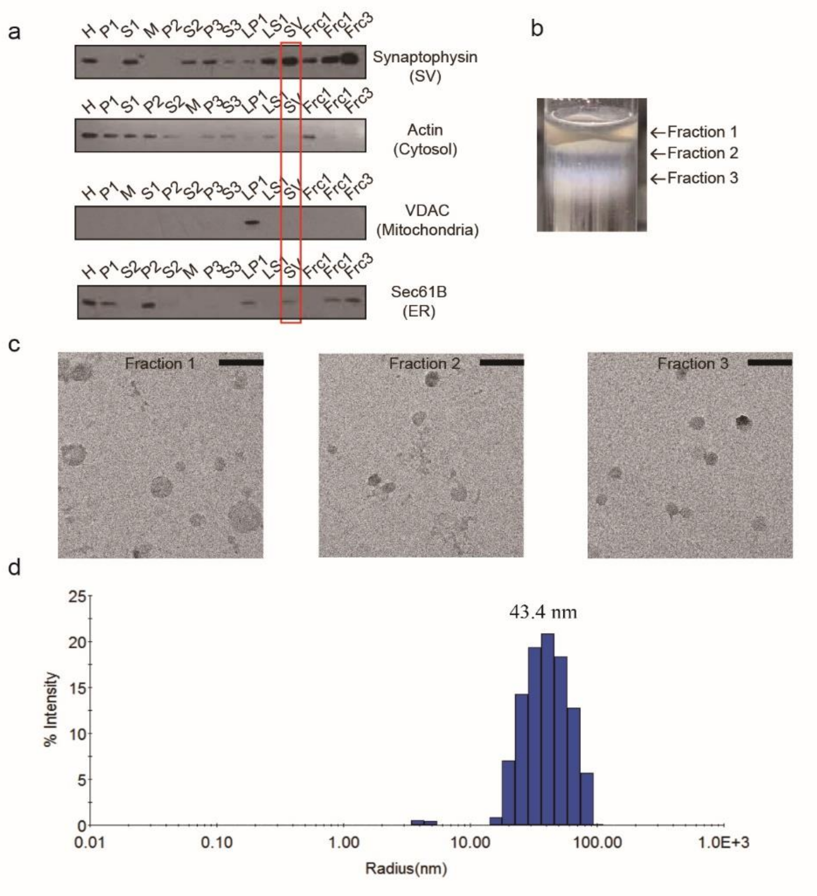

Figure S4. Mouse SV purification.

a, Western blot of fractions collected from each step of SV purification. The fraction containing SV is highlighted with red box. Other fractions are labeled in abbreviations (see details in Methods). The fractions were immunoblotted for synaptophysin (SV marker), actin (cytosol marker), VDAC (mitochondria marker) and Sec61B (endoplasmic reticulum marker), respectively. M represents protein marker.

b, Fraction SV of (**a**) was further fractionated into three fractions by OptiPrep density gradient centrifugation.

c, The three fractions of (**b**) were visualized by negative staining TEM. Fraction 3 contains relatively homogenous SVs with ~50 nm in diameter. The scale bars are 200 nm.

d, Dynamic light scattering of isolated SVs.

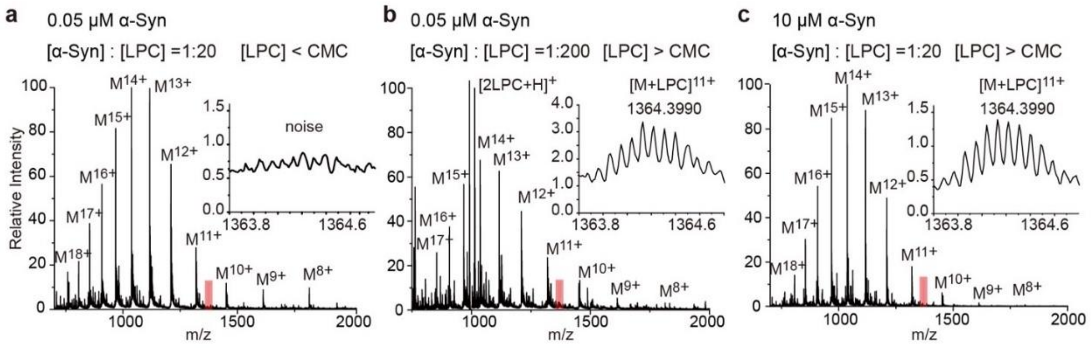

Figure S5. Characterization of LPC binding with α-syn by native MS.

ESI-MS spectra of α-syn incubated with LPC in the forms of monomer (**a**) and micelle (**b** and **c**). The critical micelle concentration (CMC) of LPC is 4-8 μM. The inserts are the zoom-in views of the MS peaks highlighted in red that correspond to the 11^+ charged complex of α-syn and LPC. The MS spectra show that α-syn binds to LPC micelles, but not monomers.

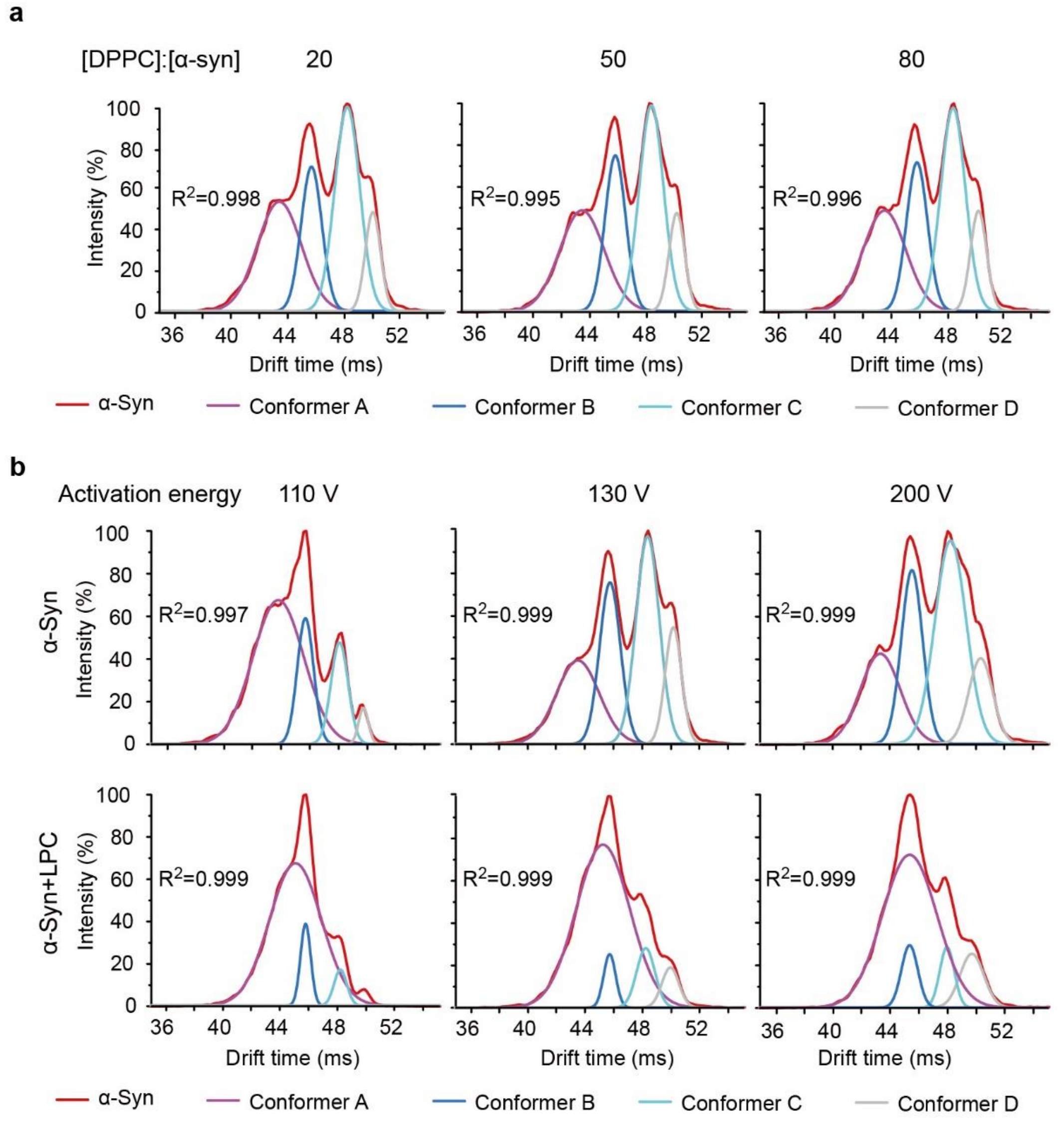

Figure S6. Conformational conversions of α-syn by IM-MS.

a, IM-MS spectra isolated from 11^+ charged α-syn upon titration of different concentrations of DPPC were shown (red line). Data were deconvolved into four conformers (A-D) by a Gaussian function fitting. R^2 values are provided. The CCS of each conformer was calculated by using a CCS calibration curve.

b, Stability of α-syn as increasing the activation energy with or without LPC. IM-MS spectra isolated from 11^+ charged α-syn were deconvolved, as increasing the activation energy for collision-induced unfolding measurement, for α-syn alone and α-syn with LPC. R^2 values are provided.

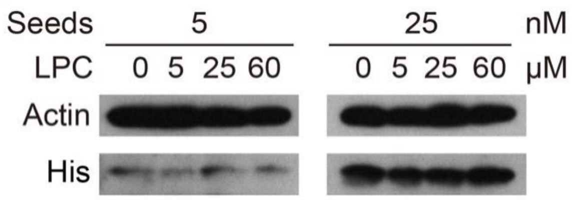

Figure S7. The effect of LPC on the cellular uptake of α-syn fibril seeds.

The C-terminal His-tagged α-syn seeds and different concentrations of LPC were added to the HEK 293T cell culture. Western blot shows the amount of seeds that entered the cells by immunoblotting for His-tag. Actin was used as a loading control.

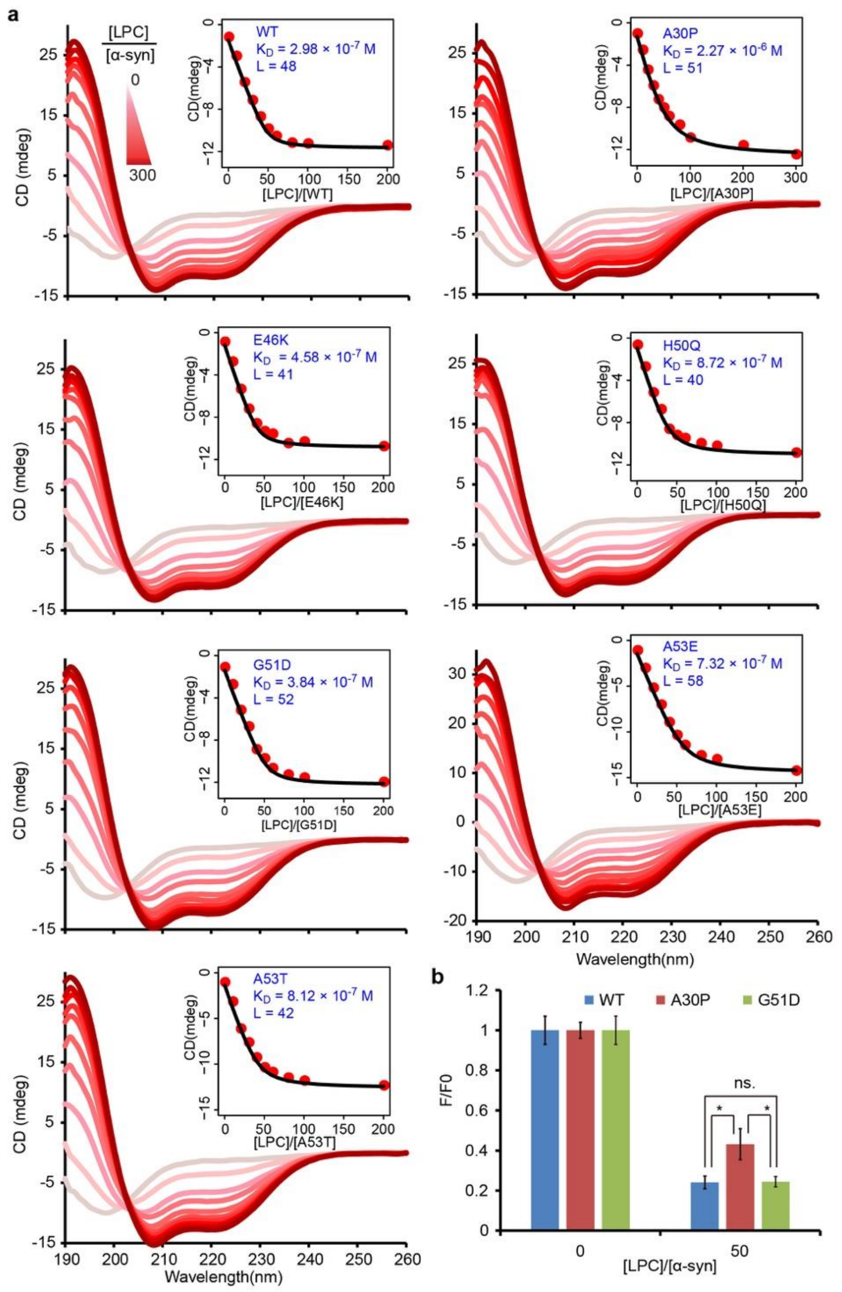

Figure S8. Binding affinity of LPC with WT α-syn and the six PD familial mutants.

a, Secondary structure changes of α-syn variants as titrated with gradient concentrations of LPC. The fitting curves were shown in the insert of each panel. The dissociation constant (K_D) and stoichiometry (L) of LPC to α-syn were calculated by using a single-step binding model for curve fitting. **b,** Effects of LPC on the fibril formations of α-syn WT, A30P, and G51D at the LPC/α-syn ratio of 50:1 by the ThT assay. The inhibitory effect of LPC on A30P α-syn significantly reduced. Data mean ± SD (n=3). * *p*-value<0.05; n.s. represents "not significant"; Student's t-test.

Methods

Animals

8 to 12-week-old male Sprague-Dawley rats (SLAC, Shanghai) and 8-week-old male C57BL/6 mice (Lingchang, Shanghai) were used in this study. The experiments were followed by the protocols approved by the Animal Care Committee of the Interdisciplinary Research Center on Biology and Chemistry, Chinese Academy of Sciences.

Cell Culture

SH-SY5Y and HEK 293T Cells have been authenticated by STR method and detected to confirm no mycoplasma contamination. SH-SY5Y cells were maintained in F12/MEM supplemented with 10% FBS (fetal bovine serum), and 1% antibiotics (penicillin-streptomycin). HEK 293T cells were cultured in Dulbecco's modified Eagles's medium replenished with 10% fetal bovine serum and 1% antibiotics (penicillin-streptomycin). All the components of medium culture were purchased from Gibco.

Lipids

Lipids were purchased from Avanti Polar Lipids.

LPC, 1-palmitoyl-2-hydroxy-sn-glycero-3-phosphocholine, Cat# 855675;

DOPC, 1,2-dioleoyl-sn-glycero-3-phosphocholine, Cat# 850375;

DOPS, 1,2-dioleoyl-sn-glycero-3-phospho-L-serine, sodium salt， Cat# 840035;

DPPC, 1,2-dipalmitoyl-sn-glycero-3-phosphocholine， Cat# 850355;

PC (15:0/18:1-d7), 1-pentadecanoyl-2-oleoyl(d7)-sn-glycero-3-phosphocholine, Cat# 791637

PE (15:0/18:1-d7), 1-pentadecanoyl-2-oleoyl(d7)-sn-glycero-3-phosphoethanolamine, Cat# 791638;

PG (15:0/18:1-d7), 1-pentadecanoyl-2-oleoyl(d7)-sn-glycero-3-[phospho-rac-(1'-glycerol)], Cat# 791640;

PS (15:0/18:1-d7), 1-pentadecanoyl-2-oleoyl(d7)-sn-glycero-3-phospho-L-serine, Cat# 791639;

PI (15:0/18:1-d7), 1-pentadecanoyl-2-oleoyl(d7)-sn-glycero-3-phosphoinositol, Cat# 791641;

PA (15:0/18:1-d7), 1-pentadecanoyl-2-oleoyl(d7)-sn-glycero-3-phosphate, Cat# 791642;

LPE (18:1-d7/0:0), 1-oleoyl(d7)-2-hydroxy-sn-glycero-3-phosphoethanolamine, Cat# 791644;

LPC (18:1-d7/0:0), 1-oleoyl(d7)-2-hydroxy-sn-glycero-3-phosphocholine, Cat# 791643;

Cer (d18:1-d7/15:0), N-pentadecanoyl-D-erythro-sphingosine(d7), Cat# 860681;

SM (d18:1/18:1-d9), N-oleoyl(d9)-D-erythro-sphingosylphosphorylcholine, Cat# 791649;
LPS (17:1/0:0), 1-(10Z-heptadecenoyl)-2-hydroxy-sn-glycero-3-[phospho-L-serine], Cat# 858141;
LPS (18:1/0:0), 1-oleoyl-2-hydroxy-sn-glycero-3-phospho-(1'-rac-glycerol), Cat# 858125.

Preparation and characterization of recombinant α-syn monomer

DNA sequences encoding human α-syn (wild type and C-terminal His-tagged) were inserted into vector pET22. The familial PD mutations of α-syn A30P, E46K, H50Q, G51D, A53E and A53T were generated by the Quick Change site-directed mutagenesis kit. All constructs were expressed in *E. coli* strain BL21(DE3) (TRANSGEN BIOTECH, Cat# CD601). The induction and the purification of α-syn monomer was followed in a previous report [1]. Bacteria were harvested by centrifugation after induction of α-syn over-expression by 1 mM isopropyl-1-thio-D-galactopyranoside (IPTG) at 37 °C for 4 h. The pellets were resuspended and lysed in the bacteria lysis buffer (100 mM Tris-HCl, pH 8.0, 1 mM EDTA, 1 mM phenylmethylsulfonyl fluoride (PMSF)). After centrifugation at 16,000 *g* for 30 min, the supernatants were boiled for 10 min followed by centrifugation at 16,000 *g* for 30 min to move the pellets. The supernatants were then stirred on ice with the addition of 20 mg/ml streptomycin for 30 min and centrifuged at 16,000 *g* for 30 min. The pH value of supernatants was adjusted to 3.5 by 2 M HCl, followed by 30-min centrifugation at 16,000 *g*. Then the supernatants were dialyzed in a buffer containing 25 mM Tris-HCl, pH 8.0 overnight at 4 °C. The dialyzed solution was injected in a Q column (GE Healthcare, Cat# 17-5156-01) and eluted by a gradient of NaCl (0-1 M), followed by further purification by a size exclusion chromatography Superdex 75 (GE Healthcare, Cat# 28-9893-33) in the buffer of 25 mM Tris-HCl, pH 8.0.

Preparation of control proteins

His-tagged control proteins including TrpEG and nsLTP (fused with trx) were purified by affinity chromatography and followed purified by size exclusion chromatography.

Metabolite extraction for *in vitro* profiling from rat brains and SH-SY5Y cells

Frozen rat brain tissues were weighted and homogenized (Bertin Technologies, Precellys 24) in pre-cold H_2O (200 µl H_2O per 10 mg tissues). 800 µl of MeOH/ACN (1:1, v/v) were added into 200 µl of homogenized solution, and vortexed for 30 s followed by incubation in liquid nitrogen for 1 min. The frozen sample was thawed at room temperature (RT) followed by

sonication for 10 min. The freeze-thaw-sonication cycle was repeated three times. To precipitate proteins, the sample was incubated for 1 h at -20°C, followed by 15-min centrifugation at 13,000 g at 4°C. The supernatants were evaporated in a vacuum concentrator (LABCONCO, 7310038). Metabolites extraction from whole cells including SH-SY5Y cells was prepared following the same protocol without the homogenization step.

For *in vitro* pull-down assay, dry metabolite extracts from rat brains and SH-SY5Y cells were re-dissolved in DMSO and then diluted with a buffer containing 25 mM Tris-HCl, pH 7.4, 100 mM NaCl to a final concentration of 2% v/v DMSO.

***In vitro* pull-down assay**

70 μM of 200 μl His-tagged α-syn, control proteins (TrpEG and nsLTP) or blank group (no protein) were incubated with 50 μl Ni-sepharose beads for 2 h at 4 °C on a rotator. Unbound proteins were removed by washing with the Tris buffer containing 25 mM Tris-HCl, pH 7.4, 100 mM NaCl. 200 μL of metabolite extract from rat brains or SH-SY5Y cells were incubated with the beads for 2 h at RT. Unbound metabolites were removed by washing with the Tris buffer. The bound metabolites were extracted by using 400 μl (200 μl each time for twice) MeOH. The extraction was dried under vacuum. The dry metabolites samples were resuspended in 100 μl MeOH before injected to LC-MS/MS for metabolite profiling. At least five biological replicates were prepared for each protein and blank control.

Liposome preparation

Lipid molecules DOPC, DPPC or DOPS were dissolved in chloroform and then evaporated under nitrogen stream. The dried lipid film was resuspended, vortexed and incubated in a water bath at 65 °C for 30 min. Then, the liposomes were vortexed and extruded 41 times through polycarbonate membrane (50 nm or 100 nm, Whatman Nucleopore Track-Etch) by an extruder apparatus (Avanti Polar Lipids, Cat# 610000).

CD spectroscopy and data fitting

WT α-syn and α-syn mutants (10 μM) were incubated with indicated concentrations of LPC in 25 mM Tris-HCl, pH 8.0, respectively. For studying the effects of salts on lipid-α-syn binding, 10 μM α-syn was incubated with 1 mM LPC or DOPS in 25 mM Tris-HCl, pH 8.0 in the presence of different concentration of NaCl (10 mM, 0.1 M, 0.5 M, 1M) or $MgCl_2$ (0.1 mM, 1 mM, 10 mM, 50 mM), respectively. The total volume of each sample is 100 μl. The path length of the quartz cuvettes used was 0.5 mm. CD spectra were measured by Chirascan (Applied

Photophysics) at 25 °C. The baseline was corrected by the blank (buffer only). CD spectra were scanned from 260 nm to 190 nm with a scanning speed of 0.5 s/nm and a bandwidth of 1 nm. Each spectrum was averaged from three biological repeats. CD signals of α-syn measured at 222 nm were plotted as a function of lipid/α-syn (molar ratios). The dissociation constant (K_D) and stoichiometry (L) of each individual lipid to α-syn were calculated by using a previously reported single-step binding model for curve fitting [2]. CD data were fitted by using Eq.2 as follows.

$$X_B = \frac{CD_{obs}\text{-}CD_F}{CD_B\text{-}CD_F} = \frac{\left([\alpha-syn]+\frac{[lipid]}{L}+K_D\right)-\sqrt{([\alpha-syn]+\frac{[lipid]}{L}+K_D)^2-\frac{4[lipid][\alpha-syn]}{L}}}{2[\alpha-syn]} \quad \text{(Eq. 1)}$$

Negative-staining transmission electron microscopy (TEM)

Each protein sample (5 μl) was pipetted on a fresh glow discharged copper grid (300 mesh) with carbon-coated. 3% uranyl acetate was used for negative staining after washing the grid twice with H_2O. The extra uranyl acetate solution was removed by blotting, then the grid was air-dried. TEM images were obtained by a FEI electron microscope at 120 kV equipped with a LaB_6 gun and an FEI BM Eagle CCD camera.

Electroporation of α-syn into HEK 293T cells

The Neon transfection system kit (Invitrogen MPK10025) was used for electroporation. Purified α-syn was lyophilized and re-dissolved in Buffer R to reach a final concentration of 500-600 μM. HEK 293T cells were harvested by centrifugation after trypsinization and washed with PBS for three times. Cells were then resuspended with α-syn solution to a cell density of 4×10^7 cells per ml. Electroporation was conducted by the Neon transfection system. Cell-protein mixture (100 μl) with a pulse program of 1,400 V pulse voltage and 20 ms pulse width were used. Cells were then transferred into dishes with pre-warmed medium and then cultured for 5 h for cell recovery. Cells were then collected and washed with PBS for five times. The concentration of electroporated α-syn in HEK 293T cells was ~40 μg/10^7 cells.

Immunoprecipitation for in-cell and *in vivo* pull-down

2×10^7 α-Syn-electroporated cells were homogenized in a pre-cold lysis buffer (50 mM Tris-HCl pH 7.4, 150 mM NaCl, 1 mM EDTA, 1 mM EGTA, 5 mM NaF, protease inhibitors cocktail, 1 mM PMSF) and incubated with α-syn antibody (Abcam, Cat# ab138501; 1:50) at 4 °C overnight. α-Syn antibody (Cell Signaling Technology, Cat# 2642S; 1:50) was used for rat

brain samples. The same amount of Rabbit IgG (Cell Signaling Technology, Cat# 2729S) was used as a control. Then, the samples were incubated with protein A agarose beads for 4 h. Followed by washing with the lysis buffer for five times, the samples were examined by western blot and LC-MS. For LC-MS, samples were mixed with 200 μl 100% MeOH (100 μl each time for twice) to elute metabolites for in-cell profiling, 200 μl DCM/MeOH (v/v=1:1) (100 μl each time for twice) for in vivo profiling hydrophobic lipids. The extraction was dried under vacuum. The dry metabolites samples were resuspended in 100 μl MeOH for in-cell profiling or 100 μl DCM/MeOH (v/v=1:1) for in vivo profiling before being injected into LC-MS/MS for profiling hydrophobic lipids. Three biological replicates were measured for HEK 293T cell samples and at least five biological replicates were measured for rat brain samples.

LC-MS/MS analysis for metabolite profiling

The LC-MS/MS analyses were conducted by an ultra-high-performance liquid chromatography (UHPLC) system (Agilent Technologies, 1290 series) coupled with a quadrupole time-of-flight mass spectrometer (Sciex, TripleTOF 6600). For *in vitro* and in-cell profilings, chromatographic separation process were performed on Waters ACQUITY HSS T3 columns (particle size, 1.8 μm; 100 mm × 2.1 mm) at 25 °C. The mobile phases A = 0.1% FA in water (positive mode) or 0.5 mM NH_4F in H_2O (negative mode), and B = 0.1% FA in ACN (positive mode) or 100% ACN (negative mode) were used. The linear gradient eluted from 1% B for 1.5 min, 1% B to 99% B (1.5-13 min), 99% B (13-16.5 min), 99% B to 1% B (16.5-16.6 min), then stayed at 1% B for 3.4 minutes to equilibrate the column. The flow rate was 0.3 ml/min with 20 min total elution time for each analysis. For analysis, a data-dependent acquisition (DDA) method is used for data acquisition, in which, one cycle consists of one rapid TOF MS survey scan (200 ms) followed by the consecutive acquisition of 6 MS/MS spectra (50 ms per spectrum). The collision energy (CE) was 35 V and the CE spread was set as 15 V for both positive and negative modes. For *in vivo* profiling, chromatographic separations were performed on a Phenomenex Kinetex C18 column (particle size, 1.7 μm; 100 mm × 2.1 mm) at 55 °C. The mobile phases A containing 10 mM ammonium formate in H_2O:ACN (6:4, v/v) and B containing 10 mM ammonium formate in IPA:ACN (9:1, v/v), were used for both ESI positive and negative modes. Linear gradient elution was conducted from 40% to 100% B (0-12 min), 100% B (12-14 min), 100% to 40% B (14-14.2 min), then equilibrated at 40% B until 18 min. The flow rate was set as 0.3 ml/min. Each acquisition cycle consisted of one rapid TOF MS survey scan (200 ms) followed by the consecutive acquisition of 9 product ion scans (50 ms each). The CE was set as 45 V, and CE spread was set as 25 V,

for both positive and negative modes.

Metabolite identification and relative quantification

The metabolite identification and relative quantification were similar to a previous report [3]. In detail, LC-MS/MS raw data (.wiff) files were converted to the mzXML format using ProteoWizard 3.0.6428, and processed by XCMS 1.41.0 [4] for peak detection and alignment. R package CAMERA was used for peak annotation after XCMS data processing [5]. Further, peak annotation was achievable through accurate mass match and MS/MS spectra match against reference libraries. First, an accurate mass match with a mass tolerance of ±5 ppm was conducted to search all lipid species in the library. Next, the corresponding experimental MS/MS spectrum was used to calculate the similarity with the MS/MS spectrum in the library for each matched lipid. An in-silico predicted lipid MS/MS spectral database referenced from LipidBlast was used for lipid identification [3, 6], and an in-house metabolite MS/MS spectral database derived from 843 standards and a commercially available database (NIST17, http://chemdata.nist.gov) were used for metabolite identification. Here a MS/MS spectral similarity score was calculated using the following dot product function (Eq.2) [7].

$$\text{Similarity Score} = \frac{\sum(([\mathrm{I}]_{\mathrm{D}}^{\mathrm{n}}[\mathrm{mz}]_{\mathrm{D}}^{\mathrm{m}})([\mathrm{I}]_{\mathrm{E}}^{\mathrm{n}}[\mathrm{mz}]_{\mathrm{E}}^{\mathrm{m}}))}{\sqrt{\sum([\mathrm{I}]_{\mathrm{D}}^{\mathrm{n}}[\mathrm{mz}]_{\mathrm{D}}^{\mathrm{m}})^2\sum([\mathrm{I}]_{\mathrm{E}}^{\mathrm{n}}[\mathrm{mz}]_{\mathrm{E}}^{\mathrm{m}})^2}} \qquad \text{(Eq. 2)}$$

Where mz and I refer to the mass-to-charge value and intensity from the database (D) or experimental (E) data, respectively, meanwhile m and n represent the weight of mz and intensity, respectively. Here, we set m = 1 and n = 0.6 (positive mode) or 1 (negative mode), which has been systematically optimized for lipids in previous work [3].

The similarity score ranges from 0 to 1, referring to no similarity and a perfect match, respectively. Metabolite matches with a score larger than 0.8 was kept as candidate. Considering mass spectrometry is difficult in distinguishing all kinds of lipid isomers, we annotated lipids in the form of the sum composition (such as PC (34:1)) or the domain consistent (such as PC (16:0_18:1)).

Relative quantification was achieved by comparing the peak area between the sample groups and the blank group. Fold changes were calculated using the mean values of the sample and blank groups. *q*-Values were calculated by Student's t-test followed by FDR correction.

Isolation of synaptic vesicles from mouse brains

Synaptic vesicles (SV) were purified following a previously reported protocol [8]. Further

purification for homogenous SVs was applied by using synaptic vesicles isolation kit. The experiment was performed at 4°C or on ice with pre-cooled reagents. Four brains from 8-week-old C57BL6 mice (male) were homogenized (900 r.p.m. for 10 min) in 25 ml 4 mM HEPES-Na, pH 7.4 and 320 mM sucrose buffer (HB) with protease inhibitors by using a PTFE pestle in a 40 ml glass tube (Sigma, P7984). The homogenates (**H**) were centrifuged at 1,500 *g* for 10 min. The supernatants (**S1**) were collected and kept on ice. The pellets (**P1**) were resuspended with 25 ml HB and homogenized (900 r.p.m. for 10 min), followed by centrifugation at 1,500 *g* for 10 min. Then, the pellets (**P2**) were discarded. The supernatants (**S2**) combined with **S1** were centrifuged at 20,000 *g* for 20 min. The supernatants (**S3**) were then discarded. The pellets (**P3**) which contain synaptosomes were resuspended with 2 ml ice-cold HB and then homogenized in 20 ml H_2O at 1,200 r.p.m. for 10 min followed by adding 50 µl of 1M HEPES-Na, pH 7.4 and protease inhibitors. The homogenates were placed on ice for 30 min, and centrifuged at 20,000 *g* for 20 min. The pellets (**LP1**) were discarded. The supernatants (**LS1**) were ultracentrifuged at 70,000 *g* for 45 min. Then the supernatants were discarded. The pellets were synaptic vesicle (**SV**) extract.

In order to obtain homogenous SVs, fraction **SV** was homogenized in 10 ml storage buffer (SB) (50 mM HEPES-K, pH 7.4, 150 mM KCl) using a PTFE pestle in an 8 ml glass tube (Sigma, P7859) at 1,200 r.p.m. for 10 min followed by dilution in 10 ml OptiPrep Density Gradient Medium with vigorous vortex. The mixture was divided equally into two 13.5 ml ultracentrifuge tubes (14 mm × 89 mm) with addition of 2 ml 40% OptiPrep solution followed by centrifugation at 240,000 *g* for 6 h. Three layers appeared in the tubes. The upper (fraction 1, Frc1) and middle (fraction 2, Frc2) layers contain relatively heterogeneous SVs. The bottom layer (fraction 3, Frc3) contains relatively homogenous SVs.

Fractions of each step were diluted and normalized to a total protein concentration of 50 µg/ml for western blot. Antibodies used in this experiment were synaptophysin (Sigma, Cat# S5768; 1:1,000) for SVs, VDAC (Cell Signaling Technology, Cat # 4661,1:1,000) for mitochondria, Sec61B (Cell Signaling Technology, Cat# 2148S; 1:1,000) for endoplasmic reticulum and β-actin (Abcam, Cat# ab8227; 1:1,000) for cytoplasm.

Dynamic light scattering (DLS)

The purified SVs (50 µl ,50 µg/ml of total protein concentration) were detected by DLS. DLS experiments were performed by a DynaPro Nanostar instrument (Wyatt Technology). Parameters were set as follows: correlation function low cutoff was 1.5 µs; correlation function

high cutoff was 6 × 10^4 μs; peak radius low cutoff was 0.5 nm; peak radius high cutoff was 10^4 nm. The results were reported as the average of 10 consecutive autocorrelation functions (acquisition time = 5 s).

Fractionation of the synaptic vesicle membrane

Fractionation of lipid rafts from the SV membrane followed a previously published protocol [9]. Briefly, fraction **SV** from the SV isolation was suspended with 4 ml ice-cold Mes-buffer (25 mM Mes, pH 6.5, 150 mM NaCl, phosphatase inhibitors) supplemented with Triton X-100 to a final concentration of 1% v/v. The mixture was gently swung at 4 °C for 10 min, and then homogenized using a PTFE pestle in an 8 ml glass tube at 200 r.p.m. for 10 min, followed by adding 80% (w/v) sucrose in Mes-buffer with a final concentration of sucrose of 40% (w/v). The mixture was divided equally into two 13.5 ml ultracentrifuge tubes and overlaid successively with 6 ml 30% (w/v) sucrose and 2.5 ml 5% (w/v) sucrose. After centrifugation at 240,000 *g* for 6 h, 12 fractions (1 ml/fraction) were collected from the top to the bottom. The pellets were resuspended in 500 μl Mes-buffer as fraction 13.

Fractions were analyzed by western blot. Antibodies used in this experiment included flotillin-2 (Santa Cruz, Cat# sc-28320; 1:500) for lipid rafts and rabphilin-3A (Santa Cruz, Cat# sc-393197;1:500) for detergent-soluble membranes.

Quantitative lipidomic profilings for mouse brain and SVs

The lipids in mouse brains and homogenous SVs were extracted using a modified MTBE extraction method [10]. 50 μl of homogenized brain or SV solution were taken and diluted to 2000 μl in H_2O, followed by addition of 4800 μl extraction solvent (MTBE:MeOH = 5:1, v/v) was added containing 0.2 μg of PC (15:0/18:1-d7), 0.1μg of PE (15:0/18:1-d7), 0.05 μg of PG (15:0/18:1-d7), 0.02 μg of PS (15:0/18:1-d7), 0.02 μg of PI (15:0/18:1-d7), 0.005 μg of LPE (18:1-d7/0:0), 0.005 μg of LPC (18:1-d7/0:0), 0.05 μg of Cer (d18:1-d7/15:0) and 0.05 μg of SM (d18:1/18:1-d9). The samples were vortexed for 30 s, followed by 10-min sonication and 15-min centrifugation at 3,000 r.p.m. The upper organic layer (2000 μL) was collected. And an additional 2000 μL of MTBE was added to the bottom layer for re-extraction. The re-extraction process was repeated twice. The pooled organic layer was evaporated using a vacuum concentrator. The dry extract was reconstituted using 200 μL of DCM:MeOH (1:1, v/v) prior to LC-MS/MS analysis. An internal standard of deuterium labeled standard of LPE(18:1-d7/0:0) was also used for the quantification of LPG and LPS, combing with an external standard curve through the peak area ratio between LPE(18:1-d7/0:0) and LPG(18:1/0:0) or LPS(17:1/0:0).

The LC-MS/MS analysis was performed using an HPLC system (Agilent Technologies, 1290 series) coupled to a quadrupole time-of-flight mass spectrometer (AB Sciex, TripleTOF 6600). Chromatographic separations were performed on a Phenomenex Kinetex C18 column (particle size, 1.7 μm; 100 mm × 2.1 mm) at 55 °C. The mobile phases A containing 10 mM ammonium formate in H_2O:ACN (6:4, v/v) and B containing 10 mM ammonium formate in IPA:ACN (9:1, v/v), were used for both ESI positive and negative modes. Linear gradient elution was conducted from 40% to 100% B (0-12 min), 100% B (12-14 min), 100% to 40% B (14-14.2 min), then equilibrated at 40% B until 18 min. The flow rate was set as 0.3 ml/min. Data-dependent acquisition (DDA) method was used for MS/MS acquisition in untargeted lipidomics approach. Each acquisition cycle consisted of one rapid TOF MS survey scan (200 ms) followed by the consecutive acquisition of 9 product ion scans (50 ms each). The CE was set as 45 V, and CE spread was set as 25 V, for both positive and negative modes.

LC-MS/MS data were processed by XCMS 1.41.0 [4] for peak detection and alignment. Lipid identification was achievable through the MS/MS spectral match against an in-silico predicted lipid MS/MS database [3]. As for the quantification of LPG and LPS, an internal standard of deuterium labeled LPE and an external standard curve (between LPE (18:1-d7/0:0) and LPG (18:1/0:0) or LPS (17:1/0:0)) was measured. In this way, the peak area ratio between LPG/LPS and deuterated LPE were used for linear standard curves to determine the concentration of LPG/LPS in samples. As for other lipid molecular species, they were quantified by measuring areas under curve (called peak area) in comparison to the corresponding internal standards within the linear dynamic range.

Preparation of α-syn fibril seeds

For the ThT assay, 100 μM α-syn in a buffer containing 50 mM Tris-HCl, 150 mM KCl, pH 7.5, 0.05% w/v NaN_3 was agitated for 1 week. For inducing α-syn aggregation in HEK 293T cells, 500 μM α-syn or His-tagged α-syn in PBS was incubated for 1 day. All samples were incubated under constant agitation (900 r.p.m.) at 37 °C. α-Syn fibril seeds were prepared by sonication of mature fibrils on ice. The morphology of mature fibrils and fibril seeds was checked by TEM.

Thioflavin T (ThT) fluorescence assay

For LPC experiments, 50 μM α-syn were incubated with LPC in 25 mM Na_2HPO_4/NaH_2PO_4, pH 6.5, 0.05% w/v NaN_3, 50 μM ThT. For seeding experiments, 50 μM α-syn were incubated with increasing concentrations of LPC in 50 mM Tris-HCl, 150 mM KCl, pH 7.5, 0.05% w/v

NaN_3, 50 μM ThT with 0.5% (v/v) α-syn fibril seeds. All experiments were added in a NUNC 384 plate under constant agitation of 900 r.p.m. (double orbital) at 37 °C. ThT fluorescence signal was measured by a microplate reader (BMG Labtech) with an excitation wavelength of 440 nm and a recording emission wavelength of 480 nm. Each experiment contains three biological replicates and is repeated at least 3 times. At the endpoint of ThT assay, the morphology of the sample was imaged by TEM and the percentage of remaining soluble α-syn was measured by SDS-PAGE and analyzed by Image Lab Software.

α-Syn aggregation in HEK 293T cells treated by LPC or BEL

DNA sequence encoding human α-syn was inserted into vector pCAGGS with a C-terminal flag tag. HEK 293T cells were transfected with the recombinant DNA construct by using Polyjet In Vitro DNA Transfection Reagent (SignaGen Laboratories, Cat# SL100688). The amount of overexpressed α-syn in HEK 293T cells reached ~25 μg/10^7 cells after transfection for 2 days.

As for LPC treatment, HEK 293T cells were pre-treated with indicated concentrations of LPC for 15 h before the transfection of α-syn DNA construct. During the medium change at 7 h, indicated concentrations of LPC and α-syn fibril seeds were added into the culture medium. Cells were harvested 48 h after transfection. To assess the effect of LPC on the uptake of α-syn fibril seeds by HEK 293T cells, C-terminal His-tagged α-syn seeds were used to distinguish the exogenous α-syn seeds from overexpressed flag-tagged α-syn and endogenous α-syn. Each experiment contained four biological replicates.

As for BEL treatment, HEK 293T cells were pre-treated with Bromoenol lactone (BEL ; Sigma, Cat# B1552) (final concentration of 10 μM) for 15 h before the transfection of α-syn DNA construct. BEL was supplemented to the final concentration of 10 μM during medium change (7 h and 24 h after transfection). Cells were harvested 48 h after transfection. For western blot and LC-MS/MS analysis, each sample was normalized based on total protein concentration measured by BCA Protein Assay Kit (Thermo Fisher Scientific， Cat# 23225). The sum of auto-scaled intensity was used to calculate the total amount of LPLs. Each experiment contained four biological replicates.

For western blot analysis of α-syn aggregation in HEK 293T cells treated with BEL or LPC, the cells were further lysed with a lysis buffer containing 50 mM Tris-HCl pH 7.4, 150 mM NaCl, 1 mM EDTA, 1 mM EGTA, 5 mM NaF, protease inhibitors cocktail, 1 mM PMSF, and 0.1% NP-40 for 1h at 4°C, followed by centrifugation at 12,000 g for 20 min. The pellets

were washed with PBS for three times and dissolved in the denaturing buffer (50 mM Tris-HCl, pH 7.5, 150 mM NaCl, 5 mM EDTA, 1% SDS) overnight and boiled for 10 min. To reach a proper exposure condition, the loading amount of cell pellets was 9 times of the total cell lysate for LPC treatment and 30 times of that for BEL treatment. The primary antibodies used were as follows: α-syn (BD Biosciences, Cat# 610786; 1: 1,000), flag (Cell Signaling Technology, Cat# 2368S; 1: 1,000), actin (Cell Signaling Technology, Cat# 3700S; 1: 1,000) and His (TransGen Biotech, Cat# HT501; 1: 1,000). The amounts of α-syn in pellets were analyzed by Fiji (ImageJ).

Native MS analysis and IM-MS analysis for protein-lipid interaction

Freshly purified recombinant α-syn in MS compatible buffer (10 mM ammonium acetate) was injected into a quadrupole-time-of-flight mass spectrometer (Agilent Technologies, Agilent Ion Mobility Q-TOF 6560) and analyzed by microflow ESI source in positive ionization with a flow rate of 10 μl/min. LPC micelle or DPPC liposome was mixed with α-syn in different protein/LPC ratios. The mixture was incubated at RT for 10 to 30 min. The instrument parameters for native MS analysis include: gas temperature, 60 °C; drying gas, 5 l/min; nebulizer, 15 psi; Vcap, 3,500 V; TOF mass range from 300 to 3,200 Da. This experiment was used to investigate the α-syn and LPC interaction by monitoring the α-syn and LPC complex peaks in MS spectra.

For IM-MS analysis, the following instrument parameters were used: drying gas, 5 L/min; gas temperature, 60 °C; Vcap, 3500 V; nebulizer, 15 psi; TOF mass range from 300 to 3,200 Da; trap funnel RF, 130 V; high pressure funnel RF, 130 V; rear funnel RF, 150 V; drift tube entrance voltage, 1,300 V; drift tube exit voltage, 250 V; IMS cell pressure 4.02 Torr. For activation-induced unfolding experiment, both of the high-pressure funnel and trap funnel RF voltages were simultaneously increased from 110 V to 200 V. The increased RF voltage accelerates the ions such that they encounter neutral gas molecules with greater kinetic energy in the funnel, and induce the unfolding of protein structures.

To analyze the conformational changes with and without lipids binding in gas-phase, distributions of drift times were extracted for the ions with given m/z values (11^{+} charge state) followed by Gaussian fitting (origin 9.0) into four different conformers, A, B, C, D. The absolute CCS of each conformer of α-syn can be determined by acquiring reference data points (CCS, drift time) from a group of reference ions (Agilent Part No. G1969-85000; Part No. 18720263) with known CCSs, and performing a simple linear regression calibration of CCS measurement [11]. The relative abundance of the compact conformer A separated by ion-

mobility drift tube was calculated as a percentage of the total intensity of the peaks in the drift time distribution of 11^+ charge state of α-syn. All mass spectra were processed with the Agilent software IM-MS Browser B.07.01.

References

1. Li, Y., et al., *Amyloid fibril structure of alpha-synuclein determined by cryo-electron microscopy.* Cell Res., 2018.
2. Galvagnion, C., et al., *Chemical properties of lipids strongly affect the kinetics of the membrane-induced aggregation of alpha-synuclein.* Proc. Natl. Acad. Sci. U S A, 2016. **113**(26): p. 7065-70.
3. Tu, J., et al., *Absolute quantitative lipidomics reveals lipidome-wide alterations in aging brain.* Metabolomics, 2018. **14**(1): p. 5.
4. Smith, C.A., et al., *XCMS: processing mass spectrometry data for metabolite profiling using nonlinear peak alignment, matching, and identification.* Anal. Chem., 2006. **78**(3): p. 779-87.
5. Kuhl, C., et al., *CAMERA: an integrated strategy for compound spectra extraction and annotation of liquid chromatography/mass spectrometry data sets.* Anal. Chem., 2012. **84**(1): p. 283-9.
6. Kind, T., et al., *LipidBlast in silico tandem mass spectrometry database for lipid identification.* Nat Methods, 2013. **10**(8): p. 755-8.
7. Stein, S.E. and D.R. Scott, *Optimization and testing of mass spectral library search algorithms for compound identification.* J. Am. Soc. Mass Spectrom., 1994. **5**(9): p. 859-66.
8. Ahmed, S., et al., *Small-scale isolation of synaptic vesicles from mammalian brain.* Nat. Protoc., 2013. **8**(5): p. 998-1009.
9. Lv, J.H., L. He, and S.F. Sui, *Lipid rafts association of synaptotagmin I on synaptic vesicles.* Biochemistry (Mosc), 2008. **73**(3): p. 283-8.
10. Matyash, V., et al., *Lipid extraction by methyl-tert-butyl ether for high-throughput lipidomics.* J. Lipid. Res., 2008. **49**(5): p. 1137-46.
11. Kurulugama, R.T., et al., *Evaluation of drift gas selection in complex sample analyses using a high performance drift tube ion mobility-QTOF mass spectrometer.* Analyst, 2015. **140**(20): p. 6834-44.
